# Liver Injury Biomarkers in Pediatric Metabolic Syndrome: Key Biochemical Associations

**DOI:** 10.3390/metabo16030171

**Published:** 2026-03-05

**Authors:** Teofana-Otilia Bizerea-Moga, Tudor Voicu Moga, Sanja Panic Zaric, Rade Vukovic, Otilia Mărginean, Lazăr Chișavu

**Affiliations:** 1 Department XI of Pediatrics-1st Pediatric Discipline, Center for Research on Growth and Developmental Disorders in Children, ‘Victor Babeș’ University of Medicine and Pharmacy Timișoara, Eftimie Murgu Sq No. 2, 300041 Timișoara, Romania; bizerea.teofana@umft.ro (T.-O.B.-M.); marginean.otilia@umft.ro (O.M.); 2 3rd Pediatric Clinic, ‘Louis Țurcanu’ Children’s Clinical and Emergency Hospital, Iosif Nemoianu 2, 300011 Timișoara, Romania; 3 Department VII of Internal Medicine-Gastroenterology Discipline, Advanced Regional Research Center in Gastroenterology and Hepatology, ‘Victor Babeș’ University of Medicine and Pharmacy Timișoara, Eftimie Murgu Sq No. 2, 300041 Timișoara, Romania; moga.tudor@umft.ro; 4 Gastroenterology and Hepatology Clinic, ‘Pius Brînzeu’ County Emergency Clinical Hospital, Liviu Rebreanu 156, 300723 Timișoara, Romania; 5 Department of Endocrinology, Mother and Child Health Care Institute of Serbia “Dr Vukan Cupic”, 11070 Belgrade, Serbia; radevukovic9@gmail.com; 6 School of Medicine, University of Belgrade, 11000 Belgrade, Serbia; 7 Centre for Molecular Research in Nephrology and Vascular Disease, “Victor Babes” University of Medicine and Pharmacy, Faculty of Medicine, Eftimie Murgu Square No. 2, 300041 Timisoara, Romania; chisavu.lazar@umft.ro; 8 Nephrology Discipline, “Victor Babes” University of Medicine and Pharmacy, Eftimie Murgu Square No. 2, 300041 Timisoara, Romania

**Keywords:** metabolic syndrome, children, alanine aminotransferase, aspartate aminotransferase, triglycerides, hepatic steatosis, fatty liver, obesity

## Abstract

**Background**: The presence of metabolic syndrome (MetS) in children predisposes them to steatotic liver disease, with or without liver enzyme alterations. Early diagnosis of the degree of liver damage can stop the progression to more severe dysfunction. **Objectives**: This study aimed to establish the link between liver enzyme levels and triglyceride and cholesterol values in pediatric patients with obesity, grouped according to MetS status and metabolic dysfunction-associated fatty liver disease (MAFLD). **Methods**: The retrospective observational study included 261 pediatric patients aged between 0 and 18 years diagnosed with obesity, MetS, and MAFLD. Before initiating the study, approval was obtained from the hospital’s Ethics Committee. The clinical and biochemical data were collected from the patients’ histories. **Results**: Alanine aminotransferase showed a significant positive correlation with triglyceride levels in the overall cohort, which became stronger in children with MetS and was strongest in those with ultrasonographically confirmed MAFLD. Similarly, aspartate aminotransferase demonstrated a weak positive correlation with triglycerides in the overall population, which increased in patients with MetS and became strong in children with MAFLD. **Conclusions**: In children with MetS and ultrasound-diagnosed MAFLD, liver enzymes showed progressively stronger positive correlations with triglyceride levels, indicating a close link between dyslipidemia and liver damage. Associations between liver enzymes and total cholesterol further support metabolic dysregulation, rather than body mass index alone, as a key driver of pediatric steatotic liver disease and highlight the value of targeted liver enzyme assessment in children with MetS or hypertriglyceridemia.

## 1. Background

Childhood obesity is a 21st-century growing global epidemic that is raising the impact of obesity-related conditions [1]. Metabolic dysfunction-associated fatty liver disease (MAFLD) is identified as the deposition of fat in more than 5% of liver cells without excessive alcohol consumption. Currently, MAFLD appears to be the most common chronic liver disease in children and adolescents. It represents a spectrum of pathology ranging from simple fat accumulation (steatosis) to non-alcoholic steatohepatitis, which is characterized by inflammation and liver cell injury, likely transitioning to fibrosis, cirrhosis, and end-stage liver disease [2,3]. The global prevalence is estimated to be in the range of 7.6 to 13% in the general pediatric population and 34.2 to 47% in obese children. Prevalence is generally higher in males compared to females and increases with a higher body BMI [2,4]. MAFLD in children is a progressive disease associated with notably shorter long-term survival compared to the expected survival of the general population of the same age and sex [5]. Liver biopsy is very invasive and not convenient for many patients. As a result, there is an urgent requirement for non-invasive biomarkers for MAFLD. Liver enzymes ALT, AST, and GGT are widely adopted as screening tools, but they have limitations. MAFLD is increasingly identified as the hepatic manifestation of MetS, representing a complex interaction between liver fat accumulation, systemic inflammation, and metabolic dysfunction that begins early in childhood. Beyond obesity itself, early-life determinants such as birth characteristics and weaning practices, together with nutritional factors and micronutrient status, shape metabolic risk profiles in children by modulating lipid metabolism, insulin sensitivity, and antioxidant defense mechanisms [6,7]. The significant clinical correlation between MAFLD and obesity-related conditions, such as dyslipidemia and insulin resistance, along with its prevalence increasing in line with the components of MetS, determines MAFLD as an important element of this multisystem metabolic disorder [8,9]. Elevated aminotransferases, especially ALT, act as indicators of hepatocellular injury and are usually used in clinical settings to detect MAFLD in at-risk pediatric populations. ALT is closely related to hepatic fat accumulation and correlates with obesity and MetS components, including dyslipidemia, prediabetes, type 2 diabetes, and cardiovascular disease [9,10]. AST is less precise than ALT for hepatocellular injury due to its expression in other tissues, including cardiac and skeletal muscle. Elevated AST demonstrates a notable correlation with metabolic dysfunction in conjunction with ALT. In pediatric populations with elevated ALT, higher AST levels correlate with more severe hepatic histology. However, AST elevation when ALT is in the normal range may point to some other conditions like skeletal muscle injury, myocardial infarction, and acute kidney injury [9,11]. The impact of dyslipidemia on MAFLD pathogenesis remains unclear, but it is still an essential component shaping the severity of and metabolic dysfunctions evident in MAFLD [12]. The neutrophil-to-lymphocyte ratio (NLR) has emerged as a simple and accessible marker of systemic inflammation and has been investigated in pediatric obesity and steatotic liver disease as a potential indicator of metabolic and inflammatory burden. Although its role in the early stages of liver involvement remains uncertain, evaluating NLR alongside biochemical markers may provide additional insight into the inflammatory component of metabolic dysfunction.

## 2. Objectives

The aim of this study was to evaluate the relationships between liver enzymes and lipid parameters in pediatric patients with obesity, stratified by MetS and MAFLD status. The primary endpoint was the correlation between ALT and triglyceride levels in the overall cohort and within subgroups defined by MetS and MAFLD. Secondary endpoints included correlations involving AST and GGT with lipid parameters, as well as the evaluation of enzyme–lipid relationships in children without metabolic dysfunction. An exploratory secondary endpoint was the assessment of associations between liver enzymes and the NLR as a marker of systemic inflammation.

This study also aimed to investigate whether the relationship between lipid abnormalities and liver enzymes is phenotype-dependent, thereby testing the hypothesis that metabolic dysfunction, rather than obesity alone, drives early hepatic injury in children.

## 3. Materials and Methods

### 3.1. Definitions

Obesity was classified based on age-specific BMI reference guidelines from the 2000 Centers for Disease Control and Prevention (CDC) Child Growth Charts [13], as BMI values above the 95th percentile.

MetS was defined as obesity along with at least two other components from the following: arterial hypertension, systolic blood pressure greater than the 95th percentile for age; disrupted glucose regulation, indicated by insulin levels over 15 mU/L or fasting blood glucose values exceeding 110 mg/dL; dyslipidemia, characterized by triglyceride levels of more than 1.69 mmol/L, HDL cholesterol levels under 0.91 mmol/L, or total cholesterol levels over the 95th percentile. Insulin resistance was diagnosed when the Homeostasis Model Assessment for Insulin Resistance (HOMA-IR) index exceeded the 95th percentile for age [14].

Steatotic liver disease was diagnosed with an abdominal ultrasound. Steatosis was evaluated using the Ultrasound Fatty Liver Index (US-FLI), a semi-quantitative scoring system validated in pediatric cohorts that grades steatosis based on liver–kidney contrast, posterior beam attenuation, vessel blurring, gallbladder wall visibility, diaphragm visualization, and the presence of focal fatty sparing [15,16].

MAFLD in children was defined as evidence of steatotic liver disease on imaging in the presence of metabolic dysfunction. Diagnosis required ultrasound-confirmed intrahepatic fat accumulation together with at least one of the following criteria: excess adiposity, presence of prediabetes or type 2 diabetes, or evidence of metabolic dysregulation, the latter identified by the presence of two or more metabolic risk abnormalities (elevated waist circumference, hypertension, hypertriglyceridemia, low HDL cholesterol, impaired fasting glucose, or an elevated triglyceride-to-HDL ratio), adjusted for age and sex [17,18,19].

In this study, all children met the obesity criterion; therefore, ultrasound-detected steatosis was classified as MAFLD according to pediatric consensus criteria. Further detailed definitions are provided in a previous study conducted by our research group [20,21].

### 3.2. Study Design

This retrospective observational study included pediatric patients aged 0–18 years diagnosed with obesity who were admitted to the Department of Pediatric Endocrinology, Diabetes, and Metabolic Diseases at “Louis Țurcanu” Children’s Clinical and Emergency Hospital in Timișoara, Romania, between January 2015 and December 2024.

Patients were identified through the hospital’s electronic medical records and patient registries. All consecutive eligible patients meeting the inclusion criteria during the study period were screened. Clinical, anthropometric, biochemical, and imaging data were retrospectively extracted from medical files and anonymized prior to analysis.

Inclusion criteria: these included age between 0 and 18 years, diagnosis of obesity according to CDC BMI criteria, availability of biochemical data, including liver enzymes and lipid profile, and availability of abdominal ultrasound data when MAFLD assessment was required.

Exclusion criteria: obesity secondary to endocrine disorders (e.g., hypothyroidism, Cushing syndrome), genetic obesity syndromes (e.g., Prader–Willi syndrome), monogenic forms of obesity or insulin resistance, inherited metabolic or lipid disorders (e.g., familial hypercholesterolemia, MODY), and patients with alternative causes of liver enzyme elevation, including acute or chronic viral hepatitis, autoimmune liver disease, drug-induced hepatotoxicity, muscular disorders, or other known hepatobiliary diseases, were excluded to minimize confounding factors affecting aminotransferase levels.

All patients met diagnostic criteria for obesity, while MetS and MAFLD were assessed separately based on clinical, biochemical, and imaging findings.

The study protocol was approved by the Ethics Committee of “Louis Țurcanu” Children’s Clinical and Emergency Hospital, with informed consent waived due to the retrospective, anonymized design.

### 3.3. Measurements and Analytical Determinations

Obesity was diagnosed using clinical evaluation and routine anthropometric measurements. Weight (W) and height (H) were recorded and used to calculate BMI. Infants younger than 1 year were measured on dedicated infant equipment, specifically the Kern Baby Scale MBC 20K10EM with the Mechanical Height Rod MBC-A01 (KERN and SOHN GmbH, Balingen, Germany). Children older than 1 year were assessed using standing devices. Weight was obtained with the KERN MPE 250K100HM floor scale (KERN and SOHN GmbH, Balingen, Germany), and height was measured using a Harpenden wall-mounted stadiometer with a high-speed counter (Holtain Ltd., Felin-y-Gigfran, Crosswell, Pembrokeshire, Wales, UK). BMI was calculated as weight (kg) divided by height squared (m^2^) and interpreted according to age- and sex-specific reference charts [22].

Blood pressure (BP) was measured on two separate visits in a calm setting using a calibrated sphygmomanometer. Either a Precisa^®^ N or Babyphon^®^ device (Riester, Jungingen, Germany) was selected based on age and arm size. Hypertension was defined as systolic blood pressure (BP) at or above the 95th percentile for age and sex [23]. Anthropometric and BP assessments were carried out by trained resident physicians under senior supervision.

Steatotic liver disease was evaluated using abdominal ultrasound performed by trained clinicians using an Esaote MyLabX7 system (Esaote S.p.A., Genoa, Italy) equipped with a convex transducer and standard acquisition protocols.

Venous blood samples were obtained after an 8 h fasting period, which was considered appropriate and feasible across all pediatric age groups included in the study and reflects routine clinical practice in pediatric metabolic assessment. Although fasting requirements for lipid assessment remain debated, an 8-h fasting period was applied to all participants to comply with standard recommendations for glucose and insulin evaluation. Using uniform fasting conditions minimized potential postprandial variability and ensured consistent metabolic measurements across the study cohort [24,25,26]. Samples were collected in serum separator tubes (yellow cap with gel), centrifuged at 4000 rpm for 10 min, and analyzed within 12 h. Glucose, insulin, lipid profile, ALT, AST, and GGT were measured in serum using enzymatic colorimetric spectrophotometric methods on an Abbott Alinity c analyzer (Abbott Laboratories, Abbott Park, IL, USA), according to the manufacturer’s standardized protocols. Analytical performance characteristics showed intra-assay and inter-assay coefficients of variation (CV) below 5% for ALT, AST, and GGT measurements. Internal quality control procedures were performed every 8 h, and the laboratory participates in external quality assessment programs conducted four times annually. All analyses were performed in the certified laboratory of “Louis Țurcanu” Children’s Clinical Emergency Hospital, accredited according to SR EN ISO 15189 standards [27].

The oral glucose tolerance test (OGTT) was performed following ISPAD recommendations. Glucose levels were measured fasting and 2 h after administering an oral glucose load of 1.75 g/kg body weight (maximum 75 g) [28]. Blood sampling was carried out by trained nursing staff using standard aseptic venipuncture techniques.

### 3.4. Statistical Analysis

Continuous variables were assessed for normality using the Shapiro–Wilk test. Normally distributed variables are expressed as mean and standard deviation (SD), while non-normally distributed variables are expressed as the median and interquartile range (IQR). Categorical variables are presented as numbers and percentages. For statistical analysis, the *t*-test or the Mann–Whitney test was used to compare two continuous variables as appropriate, while ANOVA or the Kruskal–Wallis test was applied when comparing three or more. For categorical variables, the chi-square test was used. To evaluate the relationship between liver enzymes and lipid factors, we used the Pearson correlation and calculated the r value with its 95% confidence interval. The analysis was performed in subgroups—in patients with and without metabolic syndrome and in patients with and without MAFLD. A *p*-value of less than 0.05 was considered statistically significant. All data analyses were conducted using MedCalc^®^ Statistical Software version 23.4.5 (MedCalc Software Ltd., Ostend, Belgium; https://www.medcalc.org; 2025), accessed on 12 January 2026.

## 4. Results

Our cohort consisted of 261 children with a median age of 12 years, 53.5% males, and 53.6% with MAFLD. We divided the cohort into two groups: those with and without MetS. There were no statistical differences regarding the evaluated parameters: baseline characteristics and biological ones.

Clinical, anthropometric, hematologic, and biochemical variables were compared between participants with MetS and those without. Detailed results for each parameter category are presented in Table 1, Table 2, Table 3 and Table 4.

Table 1 reports demographic indicators and anthropometric measures, along with the proportion of subjects meeting criteria for MAFLD. Data regarding the presence of MAFLD were available for 125 patients who underwent abdominal ultrasound. Among these patients, 62 had MetS, of whom 33 were diagnosed with MAFLD, while 63 did not have MetS, of whom 34 were diagnosed with MAFLD.

Table 2 summarizes hematologic parameters, including leukocyte subsets, platelet indices, and the NLR, comparing children with MetS and those without. No statistically significant differences were observed in hematologic parameters, including leukocyte subsets, platelet indices, or the NLR, between children with and without metabolic syndrome.

Table 3 outlines the distribution of lipid profile markers across the cohort and subgroup analyses.

Table 4 synthesizes biochemical indicators of liver injury in the overall population and by MetS status.

To better characterize the liver enzymes with lipid metabolism parameters, we evaluated several correlations in the entire group and in subgroups: with and without MetS and with and without MAFLD.

Table 5 shows the correlation coefficients (r) and confidence intervals (95%CI) for relationships between liver enzymes (AST, ALT, and GGT) and lipid parameters (triglycerides, cholesterol, and HDL), as well as the NLR, analyzed in the total group and by metabolic and hepatic subgroups.

**Figure 1:**
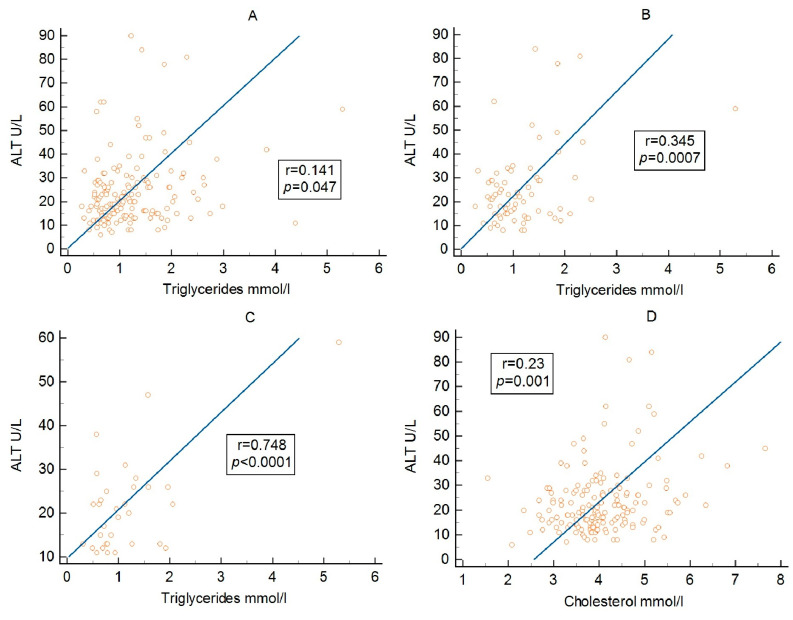
Correlations between ALT and lipid parameters: (**A**) ALT and tryglicerides in entire cohort, (**B**) ALT and tryglicerides in patients with metabolic syndrome, (**C**) ALT and tryglicerides in patients with liver steatosis., (**D**) ALT and total cholesterol levels in the overall cohort.

As shown in Table 5 and Figure 1, ALT demonstrated a weak positive correlation with triglycerides in the overall cohort (Figure 1A). A similar correlation was observed in patients with MetS (Figure 1B), whereas no significant association was found in those without MetS. In addition, ALT was positively correlated with triglycerides in patients with MAFLD (Figure 1C).

ALT also showed a weak positive correlation with total cholesterol in the overall cohort (Table 5; Figure 1D). This association remained present in both MetS and non-MetS subgroups, as well as in patients with MAFLD.

**Figure 2:**
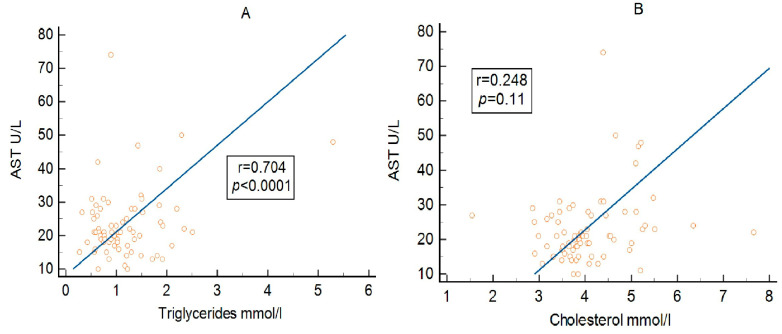
Association between AST and triglycerides (**A**) and cholesterol (**B**) in the MAFLD subgroup.

As shown in Table 5 and Figure 2, AST demonstrated a weak positive correlation with triglycerides in the overall cohort. A similar association was observed in patients with MetS, whereas no significant correlation was found in those without MetS. In contrast, AST showed a strong positive correlation with triglycerides in patients with MAFLD (Figure 2A), while no association was detected in individuals without steatotic liver disease. AST also demonstrated a weak positive correlation with total cholesterol in the overall cohort, and this relationship was present in both MetS and non-MetS subgroups (Table 5). Conversely, a positive correlation between AST and cholesterol was observed only in patients with MAFLD (Figure 2B), with no significant relationship in those without steatotic liver disease.

**Figure 3:**
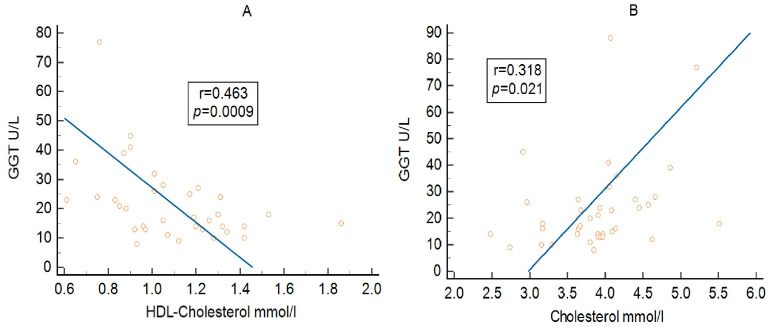
Association between GGT and HDL-cholesterol (**A**) and triglycerides (**B**) in the overall cohort.

As shown in Table 5 and Figure 3, GGT demonstrated a strong negative correlation with HDL cholesterol in the overall cohort (Figure 3A). This association was also observed in both MetS and non-MetS subgroups, as well as in patients with MAFLD.

GGT also showed a positive correlation with total cholesterol in the overall cohort (Figure 3B; Table 5), an association that was likewise present in patients with MAFLD.

As evident in Table 5, AST showed a weak positive correlation with cholesterol in the entire group, and this association was also observed in patients with and without MetS. Conversely, AST demonstrated a positive correlation with cholesterol only in individuals with MAFLD, whereas no relationship was detected in those without steatotic liver disease.

As shown in both Table 5, GGT presented a strong negative correlation with HDL cholesterol in the entire group, a correlation also present in both subgroups of patients with and without MetS and in patients with MAFLD. 

As depicted in Table 5, GGT presented a positive correlation with cholesterol in the entire group, a correlation also present in patients with MAFLD.

In both the entire study population and across all analyzed subgroups, ALT and AST showed no detectable correlation with HDL cholesterol. Additionally, none of the enzymes, including ALT, AST, or GGT, exhibited any correlation with the NLR.

## 5. Discussion

In the cohort of patients included in the study, both ALT and AST demonstrated significant positive correlations with triglyceride concentrations, progressively stronger among children with MetS and those with ultrasonographically confirmed MAFLD. These findings strengthen the theory of a close interplay between dyslipidemia and hepatic injury in pediatric obesity. When circulating triglycerides are elevated, they increase fatty acid delivery to the liver, thereby leading to intrahepatic lipid deposition, increased oxidative stress, and lipotoxic pathways. These mechanisms characterize the metabolic dysfunction leading to steatotic liver disease [29,30,31].

While ALT is already considered the most sensitive enzyme in detecting steatotic liver disease, as well as monitoring metabolic alterations in children and young adults, a rise in AST in the evolution of MAFLD generally reflects increased hepatocellular stress and inflammation with early fibrotic activity. It should be noted that we cannot strictly correlate enzyme levels with the degree of disease progression and tissue damage. However, the connection we observed between these parameters and triglyceride levels suggests a possible progression from an isolated metabolic dysfunction towards hepatic injury [31,32,33,34].

We also consider that the absence of correlation between triglyceride levels and liver enzymes in children without MetS represents an important complementary finding. This observation suggests that obesity without overt metabolic dysfunction may not be sufficient to produce measurable liver enzyme abnormalities [35]. The establishment of pediatric MAFLD requires the interconnection of several factors, such as excess adiposity, insulin resistance, and lipid metabolism disorders [9,35,36].

Our studies, correlated with investigations reported in the literature, suggest the need to diagnose and evaluate pediatric MAFLD pathology based on phenotypes, with a focus on metabolic risk, rather than solely on BMI thresholds [31,37].

In our study, ALT, AST, and GGT parameters showed weak positive correlations with total cholesterol, both in the entire sample and in the metabolic subgroups considered. We note that this observation is also confirmed by other pediatric studies that mention modest associations between elevated liver enzyme values, mainly ALT, and elevated total cholesterol and LDL cholesterol values in children presenting metabolic risk factors (obesity, family history, etc.) [12,38,39].

Notably, we identified a strong inverse correlation between GGT and HDL cholesterol across all metabolic subgroups. One possible explanation for this association is that GGT reflects oxidative stress and hepatocellular injury related to lipid peroxidation and impaired lipoprotein metabolism. Reduced HDL cholesterol levels are frequently observed in states of insulin resistance and systemic oxidative stress, conditions that also promote hepatic fat accumulation and increased GGT activity. In contrast to ALT and AST, which primarily reflect hepatocellular injury, GGT may be more closely linked to disturbances in lipid transport and oxidative pathways, which could explain its stronger inverse relationship with HDL cholesterol in children with obesity. In pediatric patients, higher GGT values consistently corresponded to lower HDL cholesterol, resulting in adverse GGT/HDL profiles, an observation also reported in previous pediatric studies. These findings support the idea that, in children with obesity and a risk of liver injury, GGT may serve as a particularly sensitive indicator of dyslipidemic changes, especially those marked by reduced HDL levels [40,41,42,43].

Increasing evidence suggests that GGT may represent not only a marker of hepatocellular stress but also a surrogate marker of insulin resistance and metabolic dysfunction. Elevated GGT levels have been associated with impaired insulin sensitivity, oxidative stress, and components of the metabolic syndrome in both adult and pediatric populations. This relationship may partly explain the strong associations observed in our cohort between GGT and adverse lipid profiles, particularly reduced HDL cholesterol [40,41].

Together, these findings support the role of liver enzymes, especially GGT, as metabolic markers that parallel cholesterol abnormalities in pediatric populations at risk for cardiometabolic and hepatic complications.

Interestingly, while liver enzymes remained modestly correlated with total cholesterol across all subgroups, their association with triglycerides disappeared in children without MetS. This divergence suggests that cholesterol dysregulation may occur earlier in the metabolic continuum, whereas triglyceride elevations may represent relatively later metabolic disturbances that become more apparent in the context of more advanced insulin resistance and cardiometabolic derangement [12,44,45]. In our cohort, triglycerides showed stronger and more consistent associations with liver enzymes than total cholesterol. This finding supports the concept that hypertriglyceridemia may represent an earlier and more sensitive marker of metabolic dysfunction and hepatic involvement in pediatric obesity. From a clinical perspective, these results suggest that elevated triglyceride levels, particularly in the context of MetS or MAFLD, may help identify children at higher risk of liver involvement, whereas total cholesterol appears to be a less specific indicator.

Integrating our findings with the existing literature, we propose a conceptual sequential model for the evolution of MAFLD, in which metabolic disturbance precedes liver injury: initially, lipid abnormalities may appear without measurable changes in enzymatic activity; subsequently, with the excessive accumulation of fat in liver cells (increased steatosis) [34]. At these stages, liver changes are generally considered reversible, and appropriate lifestyle interventions can favorably influence the course of the disease. The increased hepatocyte stress may lead to an increase in ALT levels and worsening inflammation, and/or the occurrence of fibrosis may lead to an increase in AST levels [34,46,47,48,49].

The finding that none of the liver enzymes, ALT, AST, or GGT, correlated with the NLR is consistent with previous studies, which indicate that systemic inflammation may not be prominent in the early phases of obesity-related liver disease. In our cohort, hematologic inflammatory markers, including NLR, did not differ significantly between groups. This finding may suggest that, in pediatric obesity, systemic inflammatory changes detected by simple hematologic indices are less pronounced or occur later in the course of metabolic dysfunction compared with biochemical alterations such as dyslipidemia or liver enzyme elevation. Previous studies in pediatric populations have shown that NLR tends to increase only in more advanced stages of liver disease, such as steatohepatitis or established fibrosis [50,51,52].

These findings suggest that NLR may not represent a sensitive early marker of MAFLD in children, as systemic inflammatory changes detectable by simple hematologic indices may be less pronounced in the early stages of metabolic liver involvement. Consequently, there is a need for more sensitive markers to detect this still reversible stage of the disease.

## 6. Limitations of the Study

The team that conducted this study acknowledges several limitations that should be taken into consideration. Firstly, there are some methodological limitations related to the retrospective observational design of the study that restrict causal inference. Consequently, the associations identified do not allow conclusions regarding disease pathogenesis or the establishment of temporal relationships between metabolic impairment and changes in liver enzyme levels. Non-invasive fibrosis scores used in adult diagnosis (e.g., fibrosis-4 index) could not be applied because they are not validated in pediatric populations and may lead to misclassification. Conversely, although liver ultrasound is a valuable tool for detecting steatotic liver disease in children, it does not permit an assessment of inflammatory activity or an accurate estimation of hepatic fibrosis severity. Elevated ALT or AST levels do not necessarily correlate with histological severity or fibrosis stage, as enzyme levels may fluctuate and can remain normal despite significant liver pathology. Another limitation is that the pubertal stage was not systematically recorded in the patients’ records and, thus, could not be included in the study, although its influence on insulin sensitivity, lipid metabolism, and liver enzyme levels is known. In addition, the relatively small number of patients, especially in the subgroup analysis, is a limitation of the current study, and the results should be carefully interpreted. Future studies with larger cohorts are needed to confirm our results.

Since the study was conducted in a single center, there are some limitations regarding the extrapolation of the study’s conclusions and their application to other pediatric populations. This is only possible by correlating our findings with the data reported by other research centers or medical units.

Finally, the presence of liver enzyme values within the normal range does not exclude a diagnosis of steatotic liver disease, and increased ALT or AST levels do not reflect the degree of liver damage. Therefore, the interpretation of biochemical data must be done with great discernment and must be correlated with other potentially informative aspects.

Despite these limitations, the study is particularly relevant from a clinical perspective, as it highlights the relationships between dyslipidemia and liver enzymes in pediatric patients at metabolic risk, not only from the perspective of excess weight but also of metabolic phenotype.

## 7. Conclusions

In obese children diagnosed with MetS and MAFLD based on clinical and paraclinical criteria, positive correlations were observed between liver enzyme values, particularly ALT and AST, and triglyceride levels. This relationship was stronger with increasing severity of metabolic dysfunction. These findings demonstrate a strong association between dyslipidemia and liver injury in pediatric populations at metabolic risk due to lifestyle factors such as sedentary behavior, unhealthy diet, and sleep disorders, as well as genetic and environmental influences [31]. The absence of a correlation between liver enzyme values and triglyceride levels in obese children without metabolic dysfunction suggests that excess adiposity, although a known risk factor for numerous comorbidities, is necessary but not sufficient to trigger detectable liver damage. The positive correlations between the liver enzymes ALT, AST, and GGT and total cholesterol observed across all studied subgroups, together with the strong inverse correlation between GGT and HDL cholesterol, support the role of liver enzymes as biological biomarkers for detecting lipid abnormalities. In this context, it is essential to recognize that metabolic disorders play a key role in the development of steatotic liver disease, rather than BMI alone. We therefore suggest that dyslipidemia should be considered both a cardiometabolic and a hepatic risk factor. Our findings translate into everyday clinical practice by supporting liver enzyme assessment in obese children with elevated triglyceride levels, particularly in the presence of overlapping MetS. However, enzymatic screening in the absence of metabolic dysfunction is not indicated, as it appears to have limited diagnostic utility. These observations support the MAFLD framework, which integrates metabolic context into diagnostic decision-making and may facilitate earlier identification of children at risk. Finally, normal lipid or liver enzyme values do not exclude steatotic liver disease, whereas enzyme elevations may signal early disease progression. It is essential to emphasize that progression from reversible steatosis toward more advanced forms of MAFLD could be prevented through an integrated approach, including early evaluation of metabolic and hepatic biomarkers, timely lifestyle interventions, and appropriate monitoring of high-risk patients.

## Figures and Tables

**Table 1:** Demographic and anthropometric characteristics of the study cohort and prevalence of MAFLD.

Parameter	Total *N* = 261	MetS *N* = 122 (46.74%)	Without MetS *N* = 139 (53, 26%)	*p* Value
Age years M + IQR	12 (9–14)	12 (9–14)	12 (9.75–14)	0.73
Sex % males	139 (53.5%)	65 (53.2%)	74 (53.6%)	0.95
Sex % females	122 (46.5%)	57 (46.8%)	63 (46.4%)	0.95
BMI kg/m^2^ M + IQR	29.2 (26.25–33.85)	28.8 (26.11–33.75)	29.3 (26.5–34.025)	0.59
MAFLD %	67/125 (53.6%)	33/62 (53.2%)	34/63 (54%)	0.93

**Table 2:** Hematologic parameters (×10^3^/ul) and neutrophil-to-lymphocyte ratio stratified by MetS status.

Parameter	Total *N* = 261	MetS *N* = 122 (46, 74%)	Without MetS *N* = 139 (53, 26%)	*p* Value
Leucocytes M + IQR	7.87 (6.62–9.8)	8.13 (6.78–9.71)	7.6 (6.55–9.86)	0.88
Neutrophils-N M + IQR	4.09 (3.08–5.53)	4 (3.09–5.53)	4.09 (3.06–5.53)	0.65
Neutrophils % M + IQR	53.5 (46.4–59.5)	52.75 (45.4–59.4)	53.7 (46.7–60.5)	0.28
Lymphocytes-N M + IQR	2.67 (2.18–3.35)	2.8 (2.31–3.33)	2.64 (2.1–3.35)	0.3
Lymphocytes % Mean ± SD	34.56 (8.95)	35.48 (9.42)	33.81 (8.51)	0.14
NLR M + IQR	1.5 (1.15–1.98)	1.45 (1.11–1.92)	1.56 (1.18–2.05)	0.25
Monocytes-N M + IQR	0.67 (0.55–0.82)	0.67 (0.55–0.78)	0.69 (0.57–0.85)	0.22
Monocytes % M + IQR	8.5 (7.2–9.82)	8.4 (7–9.7)	8.6 (7.3–10.02)	0.27
Platelets M + IQR	313 (269.75–356)	312.5 (256–359)	314 (271.75–355.25)	0.98
Mean Platelet Volume Mean ± SD	10.12 (0.89)	10 (0.86)	10.22 (0.9)	0.06

**Table 3:** Lipid profile characteristics of the study population.

Parameter	Total *N* = 261	MetS *N* = 122 (46, 74%)	Without MetS *N* = 139 (53, 26%)	*p* Value
Cholesterol M + IQR	3.91 (3.44–4.48)	3.85 (3.5–4.39)	3.97 (3.36–4.56)	0.65
LDL M + IQR	2.21 (1.86–2.73)	2.2 (1.74–2.59)	2.3 (1.94–2.83)	0.21
HDL Mean ± SD	1.07 (0.27)	1.07 (0.24)	1.07 (0.29)	0.99
Triglycerides M + IQR	1.03 (0.7–1.49)	1 (0.68–1.43)	1.08 (0.71–1.54)	0.55

**Table 4:** Liver enzyme levels (U/L) in participants with and without MetS.

Parameter	Total *N* = 261	MetS *N* = 122 (46, 74%)	Without MetS *N* = 139 (53, 26%)	*p* Value
ALT M + IQR	20 (15–28)	21 (15–29)	19.5 (14–27)	0.27
AST M + IQR	21 (17–26)	21 (18–27)	20.5 (17–24.5)	0.31
GGT M + IQR	18 (13.75–32)	19 (16–45)	15 (13–26.5)	0.108

**Table 5:** Correlations of liver enzymes with lipid parameters and NLR in the overall cohort and subgroups.

r and 95%CI, *p*	Total	MetS	Without MetS	MAFLD	Without MAFLD
ALT/triglycerides	0.24 (0.07–0.4), *p* = 0.0071	0.42 (0.237–0.575), *p* < 0.0001	0.09 (−0.15–0.33), *p* = 0.45	0.7 (0.506–0.831), *p* = 0.006	0.15 (−0.23–0.49), *p* = 0.43
ALT/cholesterol	0.25 (0.12–0.38), *p* = 0.0002	0.32 (0.12–0.49), *p* = 0.0015	0.19 (0.0001–0.37), *p* = 0.049	0.24 (−0.06–0.51), *p* = 0.11	0.29 (0.02–0.52), *p* = 0.03
AST/triglycerides	0.14 (0.001–0.275), *p* = 0.04	0.34 (0.15–0.51), *p* = 0.0007	−0.11 (−0.29–0.08), *p* = 0.25	0.74 (0.57–0.85), *p* < 0.0001	−0.02 (−0.29–0.24), *p* = 0.83
AST/cholesterol	0.23 (0.09–0.35), *p* = 0.0011	0.25 (0.05–0.43), *p* = 0.013	0.21 (0.02–0.38), *p* = 0.033	0.46 (0.19–0.67), *p* = 0.0017	0.17 (−0.09–0.42), *p* = 0.2
GGT/HDL	−0.46 (−0.66–−0.2), *p* = 0.0009	−0.58 (−0.8–−0.22), *p* = 0.003	−0.49 (−0.73–−0.12), *p* = 0.01	−0.78 (−0.91–−0.48), *p* = 0.0002	−0.29 (−0.67–0.21), *p* = 0.25
GGT/cholesterol	0.31 (0.−4–0.54), *p* = 0.021	0.29 (−0.11–0.61), *p* = 0.15	0.35 (−0.03–0.64), *p* = 0.071	0.56 (0.16–0.8), *p* = 0.0096	0.25 (−0.25–0.65), *p* = 0.32

## Data Availability

The original contributions presented in this study are included in the article, and further inquiries can be directed to the first and/or corresponding authors (bizerea.teofana@umft.ro, sanja.pnc@gmail.com). The raw data supporting the conclusions of this article will be made available by the authors upon request. All the data are presented in the current form of the manuscript.

## Abbreviations

The following abbreviations are used in this manuscript:

ALT	Alanine aminotransferase
AST	Aspartate aminotransferase
GGT	Gamma-glutamyltransferase
MAFLD	Metabolic dysfunction-associated fatty liver disease
HDL	High-density lipoprotein
LDL	Low-density lipoprotein
BMI	Body mass index
BP	Blood pressure
MetS	Metabolic syndrome
NLR	Neutrophil-to-lymphocyte ratio
OGTT	Oral glucose tolerance test
SD	Standard deviation
IQR	Interquartile range
